# Effectiveness of a Digital Lifestyle Change Program in Obese and Type 2 Diabetes Populations: Service Evaluation of Real-World Data

**DOI:** 10.2196/15189

**Published:** 2020-01-20

**Authors:** Iskandar Idris, James Hampton, Fiona Moncrieff, Michael Whitman

**Affiliations:** 1 Division of Medical Sciences & Graduate Entry Medicine University of Nottingham Nottingham United Kingdom; 2 Bath and North East Somerset CCG Bath United Kingdom; 3 OurPath London United Kingdom

**Keywords:** weight loss, mHealth, type 2 diabetes, OurPath, obesity, dietetics, cognitive behavioral therapy, empowerment, well-being, mobile app, behavior change, prevention, digital

## Abstract

**Background:**

The prevalence of type 2 diabetes mellitus (T2DM) and obesity is increasing, and the way people interact with health care is evolving. People traditionally access advice and support to improve their lifestyle and learn more about the self-management of T2DM in a face-to-face setting. Although these services have a strong evidence base, they have limitations for reaching specific groups of people. Digital programs could provide a new delivery model to help more people access health education and behavior change support, but long-term data supporting these programs are limited.

**Objective:**

The purpose of this service evaluation was to analyze the weight change of people who participated in OurPath (also known as Second Nature), a UK-based digital lifestyle change program, for either weight management or diabetes-related weight management and structured education at 6 and 12 months.

**Methods:**

Participants either paid to access the program privately (self-funded clients) or were referred by their general practitioner to participate in the program free of charge (funded by the National Health Service). Additional follow-up support was provided to help people to maintain lifestyle changes. To retrospectively assess potential weight loss, the analysis included data from participants who submitted weight readings at baseline and 6 and 12 months after starting the program. Changes in weight after 6 and 12 months were primary outcome measures.

**Results:**

For the 896 participants who submitted baseline and 6- and 12-month data, a significant change in mean weight of −7.12 kg (−7.50%; SD 6.37; *P*<.001) was observed at 6 months. Data from the same participants at 12 months showed a change in mean weight when compared with a baseline of −6.14 kg (−6.48%; SD 6.97; *P*<.001).

**Conclusions:**

The data presented here had several limitations, and there were too many uncertainties to make any reliable conclusions. However, these results suggest that digital lifestyle change programs could provide a new way to help people to access nutritional advice and support to achieve weight loss. Further research into digital education and coaching platforms is needed to establish their effectiveness.

## Introduction

### Background

Estimates indicate that 1 in 11 adults worldwide and more than 3 million people in England are now living with type 2 diabetes mellitus (T2DM) [1-3]. Due to the clear links between obesity and T2DM, finding a solution to the obesity crisis is critical to reducing the prevalence of T2DM and improving health outcomes [4].

There is strong evidence that lifestyle interventions focused on giving balanced nutritional advice can help people to improve their blood sugar, lipid, and blood pressure levels [4-6]. As a result of this, the current guidelines in England encourage the referral of patients who are overweight to clinical weight management programs [7]. According to a recent randomized controlled trial, the effectiveness of these programs can range from 3.26 kg to 6.76 kg weight loss at 12 months, depending on the length and intensity of the intervention [8]. Published real-world evidence is limited, but recent research by Public Health England suggests that only 2 out of 7 services in the North of England helped more than a third of participants achieve over 5% weight loss [9]. Although traditional weight loss programs have taken place in a face-to-face setting, this variance in outcomes presents an opportunity for health systems to trial new solutions for delivering weight management.

There is some existing evidence that suggests that technology can help support weight management in people living with nondiabetic hyperglycemia [10] or T2DM [11]. The Diabetes Prevention Recognition Program in the United States recognizes more than 120 organizations delivering lifestyle change programs through digital or remote channels. This widespread real-world adoption suggests that digital services that offer remote monitoring, patient engagement, and remote support could be relevant components for facilitating lifestyle change. However, the variety of programs and the data supporting them is minimal [10]. To build on this emerging evidence base for digital programs, this single-arm study has been conducted to retrospectively analyze the outcomes for OurPath (also known as Second Nature), a digitally delivered behavior change program based in the United Kingdom.

In a previous study, the OurPath program demonstrated significant weight loss in a small cohort with data available after 3 and 6 months [12]. Of the participants who enrolled in the program, 61% (42/69) had submitted a weight reading and had achieved a mean weight loss of 6.7% (*P*<.001) after 3 months. Data available for 51.72% (15/29) participants who submitted a weight reading at 6 months demonstrated a mean weight loss of 8.2% (*P*<.001). In this study, data from a new group of participants were analyzed retrospectively to validate and build upon these previous findings.

### Objectives

The objective was to investigate the weight change achieved for participants who continued to register weight readings 6 and 12 months after starting the program.

## Methods

### Program Description

**Figure 1:**
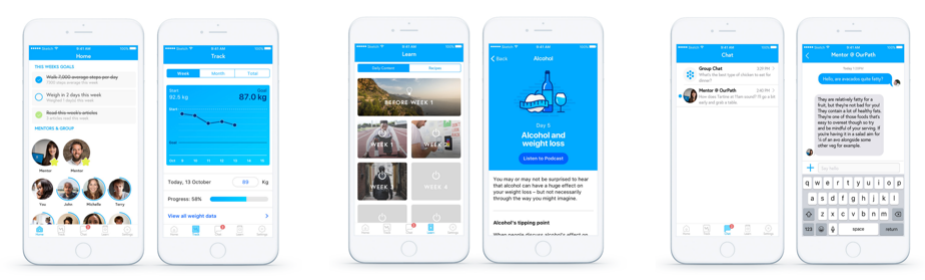
Screenshots of the digital platform.

**Figure 2:**
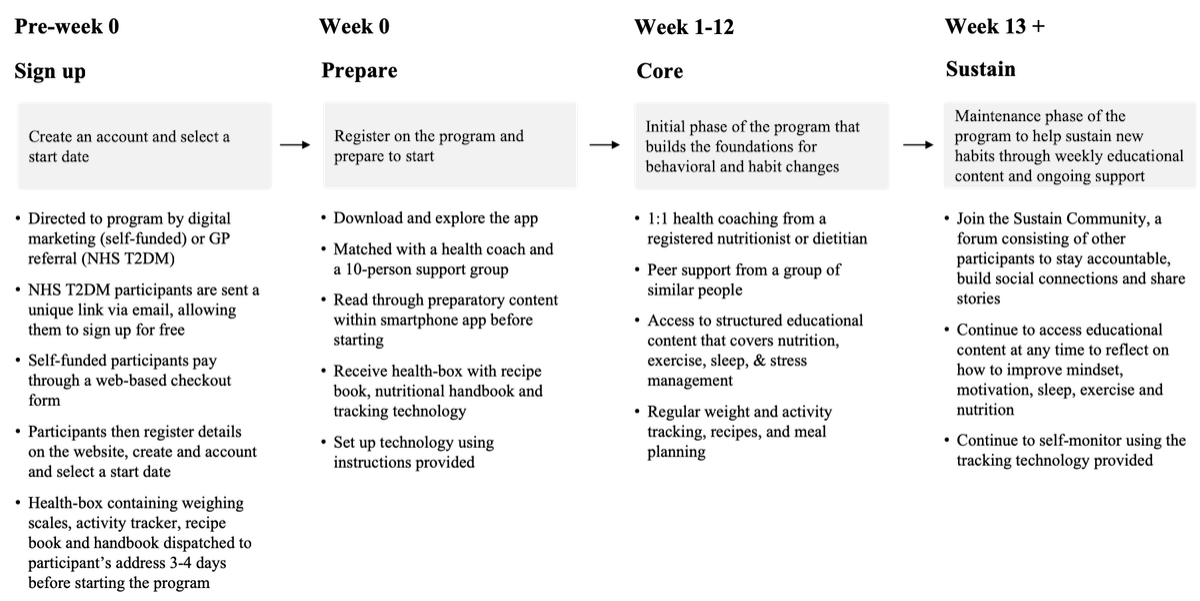
Digital program structure. GP: general practitioner; NHS T2DM: National Health Service type 2 diabetes mellitus.

OurPath is a 3-month digital behavioral change program combining one-to-one health coaching from a registered dietitian, group chat functionality with peers, structured education, and health tracking technology. All of these elements are combined within a smartphone app or Web browser–based app, accessed via compatible mobile and computer devices (see Figures 1 and 2).

The program was designed to help participants make behavioral changes while also increasing their knowledge of nutrition, physical activity, adequate sleep, and general physical and mental well-being. Several behavioral change techniques, including those outlined in the *Behaviour Change Wheel*, were incorporated into the program [13]. The adoption of new behaviors was facilitated by reducing barriers to self-monitoring changes in health (eg, diet, weight, sleep, and physical activity); incentivization through social rewards (eg, group goals and achievement badges); and nonsessional, direct support and advice from credible sources (eg, health coaches and evidence-based content) [14-16].

Registered dietitians or nutritionists delivered one-to-one health coaching via a private, text-based instant messaging function within the app. Health coaches were available between 8 am and 6 pm and replied at the beginning of the next working window for any queries submitted outside of this time frame. Messaging took place in 2 separate formats: private and group. The private chat was only viewable by the health coach and the participant, but the group chat included up to 10 other participants. The private chat allowed participants to ask specific questions and receive prompt replies on a range of topics including, but not limited to, personalized dietary requirements, negative thought patterns, and other personal health–related information.

All members within the group chat were assigned the same health coach who supervised and moderated the conversations. To maintain privacy, only the health coach and people within the group could view group messages. This feature was designed to enable participants to motivate one another, provide social accountability, and help facilitate behavior change [17]. Conversations between participants included, but were not limited to, cooking queries, nutritional debates, ingredient substitutes, and motivational support.

Participants could access educational articles with multimedia components, including plain text and video, viewed within the app. Table 1 outlines the educational themes covered during each week of the program. Each article was designed to take between 10 to 15 min to read with the ability to mark as complete when finished to track learning progress. Key educational themes covered, but were not limited to, nutrition, physical activity, stress, mental well-being, and sleep.

Every participant received a package containing a recipe book, nutritional handbook, wireless set of weighing scales, and a wearable activity tracker 3 to 4 days before starting the program. Participants and health coaches were able to view weight and daily step count within the app throughout the program. In general, participants were encouraged to register a weight reading once a week. However, the frequency of registering weight readings varied between participants, which was individualized and influenced by patient choice.

The program was divided into 2 periods: the initial phase of the program, named *Core*, and the maintenance phase, named *Sustain*. The core phase lasted for 12 weeks and was designed to be more intensive, encouraging daily engagement and primarily focusing on helping people to break pre-existing habits, form new healthier habits, and lose weight. This phase of the program was free to access for T2DM National Health Service (NHS) participants, but self-funded participants paid for the program privately. Sustain was designed to encourage weekly engagement and offer more sustainable advice, enabling people to maintain and monitor their reduced weight and healthier behaviors. During Sustain, the participants no longer had private support from the health coach, but they could still use the tracking technology and access the educational content and a forum consisting of other individuals who had completed the program, providing a supplementary level of peer support. T2DM NHS and self-funded participants both had ongoing access to the Sustain program.

**Table 1:** Educational themes explored during the digital program.

Week	Theme	Goal
0	Prepare for the program	Introduce nutritional basics around metabolism and the nutritional requirements of the body
1	Reset your lifestyle	Introduce self-monitoring of body weight and provide more detailed nutritional advice
2	Build healthy habits	Increase knowledge on sleep and physical activity
3	Tackling temptations	Introduce the role of insulin, exercise, and perceiving mistakes as learning opportunities
4	Try something new	Increase confidence in food and exercise
5	Keep your gut healthy and happy	More detailed advice on healthy nutrition, including the importance of fiber
6	Reflection on progress	Introduce self-reflection and recap on the program so far
7	How to overcome obstacles	Introduce acceptance and commitment therapy techniques and how to navigate time constraints
8	Boost your health	Further reinforcement of physical activity and nutritional advice
9	Remember—mind over matter	Advice on tackling challenging moments and fluctuating energy levels
10	Maximizing rest and relaxation time	Reinforce the importance of stress management and sleep
11	Top tips to take away	Recap on key points covered so far
12	Moving forward	Reflect on progress over the last 12 weeks and introduce sustain and develop strategies for long-term weight maintenance

To access the program, both groups of participants registered their details on the OurPath website. Each participant answered a series of questions about their current lifestyle and what changes they would like to make. After confirming that they were ready to make a change to their lifestyle, they created an account with a secure username and password and selected a date to start the program. After this registration process had been completed, participants were able to log into and access the full functionality of the app. This involved communicating with their health coach; speaking to other participants in their group; accessing educational articles; viewing recipes; and self-monitoring changes in their diet, steps, weight, and sleep.

National Institute of Health and Care Excellence guidelines, including Clinical Guideline 43 and Public Health Guideline 38, informed the advice and recommendations given by the health coaches [18,19]. This advice was focused around limiting highly processed goods while encouraging home-prepared, nutritionally balanced meals.

The program was designed and validated by a clinical advisory team consisting of diabetes specialist clinicians, general practitioners (GPs), psychologists, behavioral scientists, and registered dietitians.

This service evaluation did not require institutional review board approval as the project did not include any access to personally identifiable information. Data were routinely collected from participants, as part of the OurPath program, who had already consented for their data to be collected and anonymized for medical research purposes. For NHS T2DM participants, the program was part of usual care, and participants were not randomized to a treatment.

### Participants

Participants either paid to access the program privately (self-funded clients) or were referred by their GP to participate in the program free of charge (funded by the NHS). Self-funded participants purchased the program for help to manage their weight. All participants referred through the NHS were already living with T2DM and were invited to participate in the program for weight management and behavioral change support. All participants included in this analysis were overweight, or living with obesity, with a BMI>25 kg/m^3^.

Participants were adults, aged 18 years and older, and were living in the United Kingdom at the time of participation. Although time since diagnosis was not directly measured, it was also not set as an eligibility criterion for program participants. Medication usage was also not measured or used as an inclusion criterion.

All participants consented to their anonymized data being used for analysis and publication, taking part in the program between January 1, 2017, and August 1, 2018.

To assess the potential weight loss of people who continued to use the tracking technology, we only included participants who submitted weight readings at baseline and 6 and 12 months after starting the program.

### Measures

Participants self-reported their gender, height, and age during the Web-based registration process, but all weight readings were collected using the wireless weighing scales provided. Participants were directed to place weighing scales on a hard and flat surface. Once they had been used, the wireless weighing scales automatically transmitted weight data directly to a central database, displaying readings as a graph on the smartphone app. For the collection of these data, the scales fed readings into a weight validation algorithm, which only accepted weight readings within an expected range. This calculation accounted for the value of previous weight reading and the time since that reading was registered, automatically notifying the participant of any invalid weight readings via email. This process was designed to exclude anomalous readings and ensure the capture of consistent and objective readings for analysis. The validated weight readings registered 4 weeks before or after the data collection milestones were retrieved from the database. For this analysis, the lowest retrieved weight reading within the 8-week window was used.

### Primary Outcomes

A single-arm retrospective longitudinal study design was used to evaluate the effect on weight after having enrolled in the OurPath program. Primary outcome measures were changes in weight after 6 and 12 months. This change in weight was analyzed in kilograms and percentage reduction in initial body weight. The percentage of participants achieving more than 5% and 10% reduction in initial body weight was also included as a primary outcome measure.

### Statistical Analysis

The R open-source statistical language was used through the R-Studio interface to calculate statistical tests with *P* values and generate visual representations of the data. One-way Student *t* tests were used, with the null hypothesis being an average weight loss of 0 kg (no weight loss) and the test hypothesis that the population mean was greater than 0. *P* values reported in this publication also held significance at 95% level using a null hypothesis of 5.5 kg weight loss.

## Results

### Baseline Characteristics

**Figure 3:**
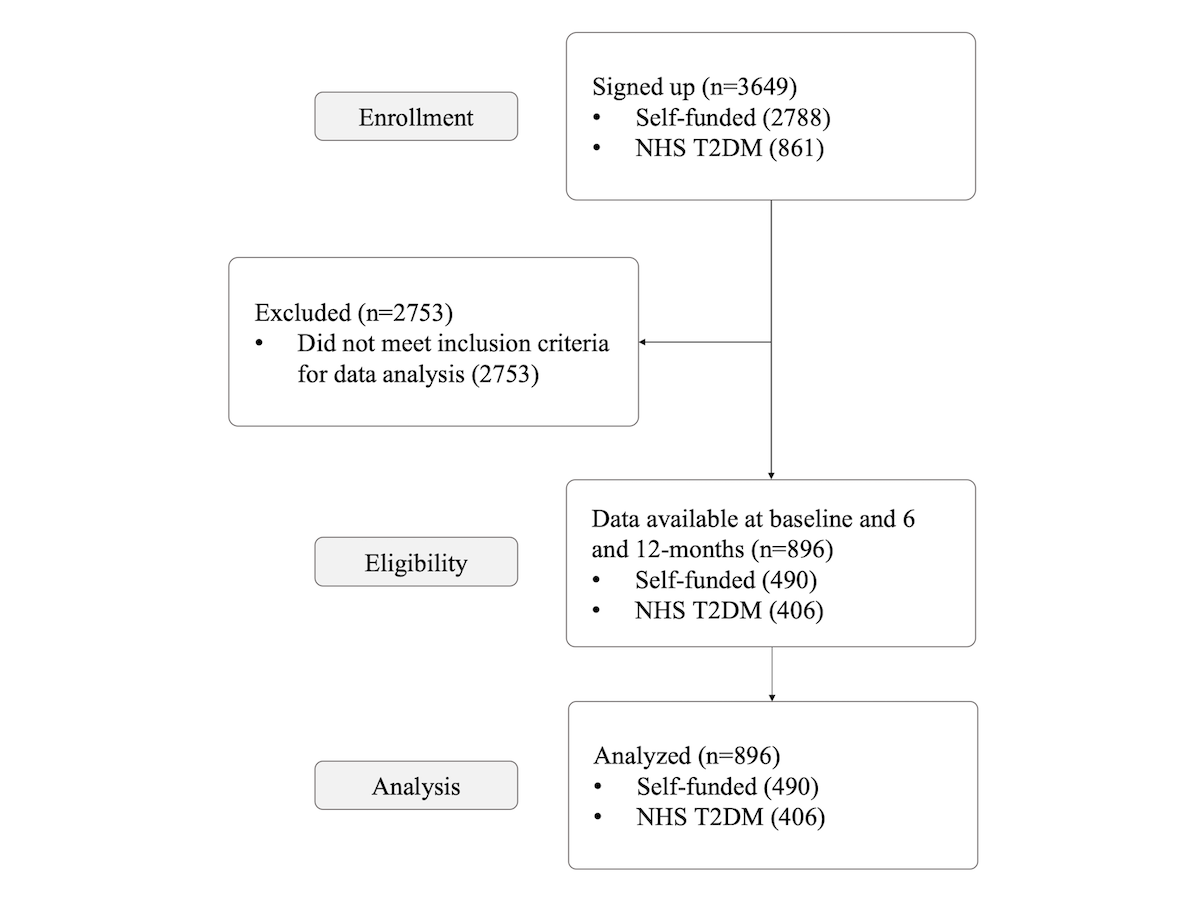
Study participant flowchart. NHS T2DM: National Health Service type 2 diabetes mellitus.

The total number of participants who took part in the program between January 1, 2017, and August 1, 2018, was 3649 (see Figure 3). Of those, 2788 were in the self-funded group, and 861 were in the T2DM NHS group.

Of the 3649 participants of the OurPath program, 896 participants submitted both 6- and 12-month weight readings, meeting the criteria for the data analysis.

Due to the retrospective nature and real-world setting of this study, it was not possible to ensure an even distribution of comparable characteristics in each cohort (see Table 2). A higher proportion of females to males took part in the program, with 70% (627/895) female and 30% (269/895) male participants. Participants had a mean starting BMI of 33.7 kg/m^2^ (SD 6.1) and starting weight of 94.7 kg (SD 18.9).

**Table 2:** Baseline characteristics of program participants.

Characteristic		Overall	Self-funded	National Health Service type 2 diabetes mellitus
Age (years), mean (SD)		49.4 (12.6)	48.5 (11.8)	51.2 (12.6)
Weight (kg), mean (SD)		94.7 (18.9)	94.5 (20.5)	95.1 (18.6)
BMI (kg/m^2^), mean (SD)		33.7 (6.1)	33.9 (6.6)	33.4 (6.3)
**Gender, n (%)**				
	Female	627 (70.0)	406 (82.9)	221 (55.4)
	Male	269 (30.0)	84 (17.1)	185 (45.6)

### Primary Outcomes

Data from the 896 participants who registered readings at both 6 and 12 months showed a statistically significant change in weight at 6 months (mean −7.1 kg, SD 6.4; −7.5%; *P*<.001) and at 12 months (mean −6.1 kg, SD 7.0; −6.5%; *P*<.001).

The proportion of people achieving more than 5% weight loss and more than 10% weight loss was also analyzed for all participants with data available at both milestones.

After 6 months, 60.1% (546/896) participants with data available achieved over 5% weight loss. This level of weight loss was achieved by 66.5% (270/406) of the T2DM NHS participants and 56.3% (276/490) of the self-funded participants.

In addition to this, 29.4% (264/896) of all the participants with data available achieved more than 10% weight loss. This level of weight loss was also seen in 31.5% (128/406) of the participants in the T2DM NHS group, and 27.8% (136/490) of the participants in the self-funded group also achieved over 10% weight loss at 6 months.

After 12-months, 53.3% (478/896) participants with data available achieved over 5% weight loss. This level of weight loss was achieved by 55.7% (226/406) of the participants in the T2DM NHS group and 51.4% (252/490) of the participants in the self-funded group. In addition to this, 23.5% (211/896) of all of the participants with data available achieved more than 10% weight loss. This level of weight loss was also seen in 24.6% (100/406) of the participants in the T2DM NHS group and 22.6% (111/490) of the participants in the self-funded group.

The data presented in Table 3 show that in the T2DM NHS group, 93.8% (382/406) of the participants lost weight at 6 months, and 87.7% (356/406) of the participants lost weight at 12 months. Of those with data available in this cohort, 5.2% (21/406) of the participants gained weight at 6 months, and 8.4% (34/406) of the participants had gained weight at 12 months.

**Table 3:** The proportion of participants with data available achieving more than 5% and 10% weight loss.

Level of weight loss achieved	6 months	12 months
Participants with data available achieving >5% weight loss (N=896), n (%)	546 (60.1)	478 (53.3)
NHS T2DM^a^ participants with data available achieving >5% weight loss (N=406), n (%)	270 (66.5)	226 (55.7)
Self-funded participants with data available achieving >5% weight loss (N=490), n (%)	276 (56.3)	252 (51.4)
Total participants with data available achieving >10% weight loss (N=896), n (%)	264 (29.4)	211 (23.5)
NHS T2DM participants with data available achieving >10% weight loss (N=406), n (%)	128 (31.5)	100 (24.6)
Self-funded participants with data available achieving >10% weight loss (N=490), n (%)	136 (27.8)	111 (22.6)

^a^NHS T2DM: National Health Service type 2 diabetes mellitus.

In the self-funded group, 90% (449/490) of the participants lost weight at 6 months, and 85.1% (417/490) of the participants lost weight at 12 months (396/460). Moreover, 4.7% (23/490) of the participants gained weight at 6 months, and 10.2% (50/490) of the participants had gained weight at 12 months.

## Discussion

### Principal Findings

**Figure 4:**
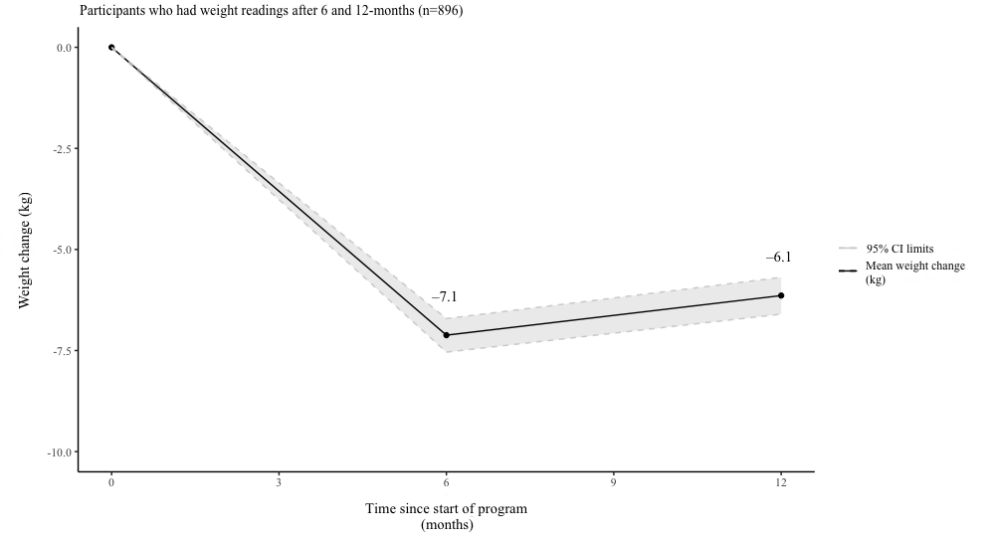
Weight change of study participants over time.

This study showed that participants who registered weight readings achieved statistically significant weight loss at 6- and 12-month milestones (see Table 4 and Figure 4). These results align with previous research and build on the emerging evidence base surrounding digital behavior change interventions [12,20]. The mean weight loss achieved at 12 months by those with data available exceeded a 5% reduction in initial body weight, which has been associated with a reduction in disease risk for T2DM [21].

**Table 4:** Weight change for participants at 6- and 12-month collection milestones.

Cohort with data available	Data collection milestone				
	Baseline weight (kg), mean (SD)	6-month weight (kg), mean (SD)	6-month weight change (kg), mean (SD)	12-month weight (kg), mean (SD)	12-month weight change (kg), mean (SD)
Consolidated participant weight change from baseline	94.3 (18.9)	87.2 (18.4)	−7.1 (6.4)	88.2 (19)	−6.1 (7)
Self-funded participant weight change from baseline	94.9 (19)	88.1 (18.7)	−6.7 (6.6)	88.9 (19.2)	−5.9 (6.8)
National Health Service t*ype 2 diabetes mellitus* participant weight change from baseline	93.7 (18.7)	86.1 (17.9)	−7.6 (6.2)	87.3 (18.7)	−6.4 (7.2)
Male participant weight change from baseline	102.2 (19.3)	93.3 (20.2)	−8.9 (7.8)	95.0 (20.8)	−7.1 (7.2)
Female participant weight change from baseline	91.8 (18)	85.3 (17.8)	−6.5 (5.7)	86.0 (18.3)	−5.8 (6.9)

When compared with the NHS T2DM group, a much higher proportion of the self-funded group were excluded from the analysis because of incomplete data. In total, 24.55% (896/3649) of the participants met the inclusion criteria for the data analysis. Of the T2D NHS participants who enrolled in the program, 47.5% (409/861) registered weight readings at 6 and 12 months compared with 17.57% (490/2788) in the self-funded group.

There could be several reasons for the disparity in data available between the groups. For certain individuals, health-related motivation could promote more self-monitoring behavior. In addition, previous research has shown that significant weight loss can also positively influence motivation, and similarly, that any weight gain can be demotivating, leading to decreased engagement [22,23]. However, it could also be argued that fewer patients chose to self-monitor their weight as the program progressed as there were fewer prompts to do so, particularly after the program had ended. Without more complete weight data, further research is needed to establish the long-term outcomes of all participants.

**Figure 5:**
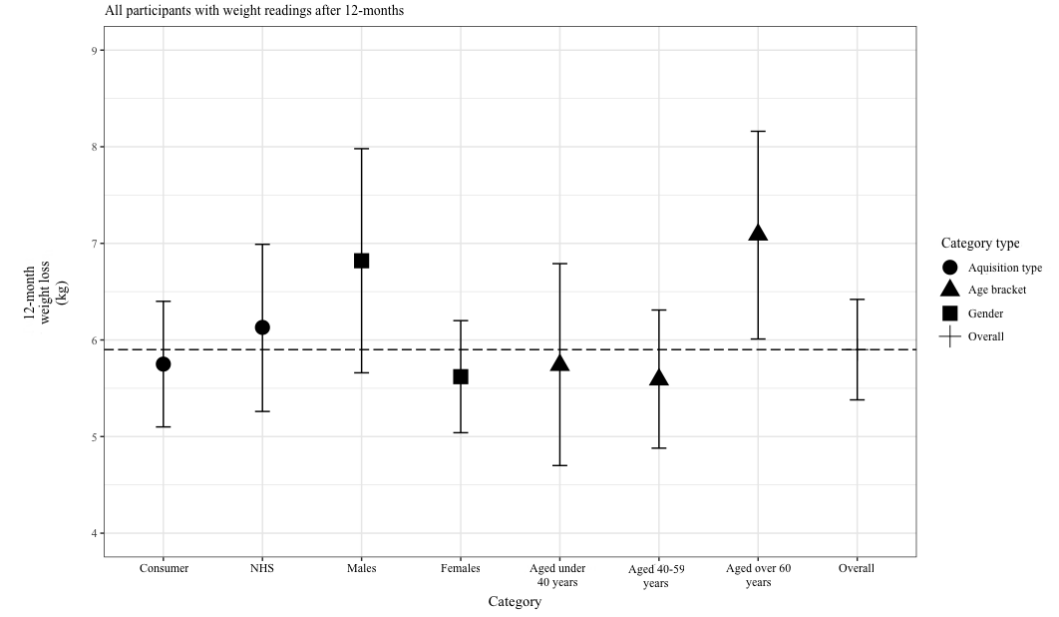
Twelve-month weight loss by demographic. NHS: National Health Service.

Of those who registered weight readings, male participants lost more weight than female participants (see Figure 5). There could be several reasons for this, eg, the male cohort analyzed had a higher mean baseline weight of 102.2 kg (SD 19.3) compared with the female mean baseline weight of 91.8 kg (SD 18.0). Although there are several differences in the hormonal balance and metabolism of adipose tissue between males and females, the reason for these results was not clear within the remit of this study [24].

Similar results were achieved in the self-funded group and T2DM NHS group, suggesting that participants from both cohorts with data available were able to achieve weight loss. Weight change also varied between age brackets, with the analyzed participants who were older than 60 years achieving more weight loss. These results align with previous research indicating that older age can be associated with increased adherence to weight loss programs [22]. In addition to differences in gender, this trend could equally warrant further investigation to establish whether there is a correlation between age and adherence to digital programs.

As this was not a randomized controlled trial, and we used a single-arm study design, we were unable to compare the results with a control or a group receiving usual care. It was also difficult to control variability as an existing dataset was used for analysis. However, similar results were seen in the treatment arm of a randomized control trial conducted in the United Kingdom, which looked at weight loss in a 12- and 52-week weight management program, with a control group of participants receiving brief lifestyle advice [8].

Randomized controlled trials present several practical challenges, and because digital technology is constantly evolving, real-world evidence can provide an important and accessible way of evaluating new apps. Even in a controlled setting, the digital nature of the program would make it difficult to blind participants to the intervention. However, this study provides real-world evidence from free-living people outside of a controlled environment, supplementing findings from randomized controlled trials and furthering the understanding of a relatively new method of providing weight management services [25].

When compared with baseline, 8.4% (34/406) of T2DM NHS and 10.2% (50/490) of self-funded participants with data available at 12 months showed some level of weight gain. This proportion of participants is small but noteworthy and indicates that continued self-monitoring is not only demonstrated by people who successfully achieved weight loss. Future studies should include other indicators of health, such as hemoglobin A_1c_ and blood pressure, which could demonstrate any broader benefits of lifestyle modification.

Implementing high-quality and cost-effective health education for people living with obesity or T2DM is a significant challenge for weight management services. In a digital setting, specialist health coaches can reach a large number of service users simultaneously, making the service efficient to maintain. Given the challenges surrounding NHS resourcing, particularly in dietetics, this method of digital delivery has the potential to cater to the vast number of people who potentially need support.

Digital methods of delivery also have the potential to impact the accessibility of structured education. The educational content delivered through remote programs is continuously accessible and can be divided into more manageable quantities, making it easier for participants to assimilate [26]. The face-to-face setting of traditional programs can make this difficult for some people, as there is variability between existing knowledge and learning speeds [27,28]. Digital interventions could enable the service user to learn at their own speed through interactive content, enabling them to seek advice from a health coach whenever necessary.

The tracking technology provided enabled people to self-monitor their progress. For some people, this immediate feedback can provide further motivation, which has been shown to lead to further reinforcement of healthy behaviors, leading to more positive outcomes [29]. The monitoring of weight and physical activity also provides useful information for the health coach, allowing them to tailor their nutritional advice, activity recommendations, and goal setting according to a participant’s progress. This level of individualization could be an advantage of digitally delivered programs, and although the results from this study did not allow for any robust conclusions, it would be interesting to investigate this further in future studies. Furthermore, the extent of the data presented did not indicate which elements of the program were the most effective for facilitating behavior change and weight loss. More research is needed to determine whether it is the regular feedback from the health coach, the continual self-monitoring, the educational articles, or a combination of the components that work best for participants.

### Limitations

As this study retrospectively analyzed real-world data, there was no control group. Without a control group, the results have limited validity and must be interpreted carefully. However, the results can be compared with a study that evaluated weight loss outcomes from another digitally delivered weight loss program. This study had a much larger sample size available for analysis but also found that users were successful in losing a significant amount of weight using a digital program. The results of this study suggest that frequent self-monitoring, weighing, and logging food and exercise resulted in more weight loss [30].

The observational setting of this study also made it very difficult to control the proportion of participants who registered weight readings. This lack of data did not allow for the effectiveness of the program to be properly evaluated.

Only data from the weighing scales were collected, and more detailed information on the usage of the program was not available. Without these data, investigating differences in completion or engagement with specific features of the program was not possible. On the basis of the research into the adherence to other programs, those who continued to register weight readings were more motivated and, therefore, more likely to have lost weight, introducing a self-selection bias to the data. However, it is also important to note that readings were still registered by 9.2% (84/896) of the participants who gained weight.

### Conclusions

The majority of people who continued to register weight readings at 6 and 12 months did achieve significant weight loss. Although the data presented had several limitations, and there were too many uncertainties to make any reliable conclusions, this study adds to existing real-world data, which suggest that digital lifestyle change programs could be a useful tool to help people to access nutritional advice and support.

## Abbreviations

GP: general practitioner
NHS: National Health Service
T2DM: type 2 diabetes mellitus
